# PTPRO is a therapeutic target and correlated with immune infiltrates in pancreatic cancer

**DOI:** 10.7150/jca.64661

**Published:** 2021-10-30

**Authors:** Xuben Hou, Jintong Du, Hao Fang

**Affiliations:** 1 Department of Medicinal Chemistry and Key Laboratory of Chemical Biology of Natural Products (MOE), Cheeloo College of Medicine, School of Pharmaeutical Science, Shandong University, Jinan, Shandong, 250012, China.; 2 Shandong Cancer Hospital and Institute, Shandong First Medical University, Jinan, Shandong, 250117, China.

**Keywords:** PTPRO, prognosis, biomarker, pancreatic cancer, tumor-infiltrating

## Abstract

As a member of protein tyrosine phosphatases (PTPs), the protein tyrosine phosphatase receptor type O (PTPRO) has attracted increasing attention for its important roles in cell signaling. Currently, the roles of PTPRO in human cancers remain elusive. Herein, we performed bioinformatic analyses and revealed the potential oncogenic role of PTPRO in specific cancer types. Further *in vitro* experiments indicated that inhibition of PTPRO suppresses the proliferative abilities of tumor cells in pancreatic cancer, blood cancer, and breast cancer. Moreover, small molecular PTPRO inhibitor could induce cell apoptosis and affect the cell cycle in pancreatic cancer. In addition, PTPRO expression promoted the infiltration of CD8+ T, macrophages, dendritic cells, and neutrophils, in pancreatic cancers. Our findings suggested PTPRO may serve as a potential drug target for pancreatic cancer.

## Introduction

Nowadays, phosphorylation of protein tyrosine, which is modulated jointly by protein tyrosine phosphatases (PTPs) and protein tyrosine kinases (PTKs), is recognized as a major regulatory mechanism of cell signaling 1. Elevated tyrosine kinase activity is frequently observed in many human cancers and most of the known dominant oncogenes are PTKs 2. Considering that PTPs catalyze the reverse reaction, it is speculated that PTPs might block this oncogenic transformation and act as tumor suppressors 3. However, numerous studies have revealed that PTPs do not necessarily oppose PTK activity but can also exert oncogenic functions 4, 5. In contrast to the PTK activity, PTPs can mediate signal transduction pathways either negatively or positively. As such, they activate or inhibit tyrosine kinases through dephosphorylation of either the kinase or its downstream target 6.

Even though targeted therapy based on PTK has progressed to the levels that they have been approved by the FDA, the development of PTP inhibitor is still in progress. Genetic assessment of various cancers in humans at a large-scale level recently revealed the value of PTPs as candidate tumor inhibitors or as potential oncoproteins. Currently, the Src homology domain-containing phosphatase 2 (SHP2) and the three-membered family of phosphatases of regenerating liver (PRL) have been identified as oncogenic members of the PTP superfamily 7-12. At present, four SHP2 inhibitors are currently undergoing clinical trials for the therapy against solid tumors. These findings shed new lights on the PTP-targeted anti-cancer therapy.

Protein tyrosine phosphatase receptor-type O (*PTPRO*) is categorized as a receptor-type PTP of the R3 subtype. Evidence indicates that PTPRO can be downregulated via methylation in some forms of tumors, such as breast cancer, hepatocellular carcinoma, lung cancer, chronic lymphocytic leukemia, and esophageal carcinoma 13-18. Nevertheless, to our knowledge no studies have systematically investigated the expression and prognostic value of PTPRO in various human cancers. Therefore, the specific objective here was to better characterize the potential functions of PTPRO in human cancers. Herein, we employed the PrognoScan 19 and Oncomine 20 databases, as well as Kaplan-Meier plotter 21 to conduct a comprehensive analysis of PTPRO expression and its link with cancer prognosis. *In vitro* tests indicated that inhibition of PTPRO impacts the cell growth, cell apoptosis as well as cell cycle in pancreatic cancer. Also, we examined the link between PTPRO expression and KEGG pathways using Gene Set Enrichment Analyses (GSEA) 22. Furthermore, we utilized TIMER 23, 24 (Immune Estimation Tumor Resource) to analyze the relationship between PTPRO and tumor-infiltrating immune cells in the various tumor microenvironments. Our findings revealed that PTPRO might play a crucial role in the progression of pancreatic cancer.

## Materials and Methods

### Bioinformatic analysis

Data on PTPRO gene expression in certain forms of cancers were retrieved from the Oncomine database 20. The PrognoScan database 19 was employed to analyze the relationship between PTPRO expression and survival in various tumor types. The threshold was adjusted to a Cox *P*-value < 0.05. Meanwhile, Kaplan-Meier plotter (http://kmplot.com/analysis/) 21 was used to analyze the association between PTPRO expression and survival in rectum and pancreatic cancers. Also calculated were log-rank P-value and hazard ratio (HR) with 95% confidence intervals. GSEA were carried out using TCGA gene expression data of pancreatic cancer samples with pearson measure and PTPRO was used as the gene phenotype. The link between PTPRO expression and tumor purity, as well as immune infiltrate abundance, such as B cells, CD8^+^ T cells, CD4^+^ T cells, neutrophils, dendritic cells, and macrophages, were analyzed via TIMER (http://timer.cistrome.org/) 23.

### Anti-proliferation assay

We conducted the sulforhodamine B assay (SRB) to assess the proliferative ability of the adherent cells. In brief, the cells were seeded into 96-well plates and subjected to various concentrations of PTPRO inhibitor **GP03**, then incubated for 72 hours. Subsequently, the cells were fixed for 1 hour in 10% trichloroacetic acid at 4 °C. After that, the cells were washed thrice in tap water then air-dried. Surviving cells were stained at room temperature with 0.4% (w/v) SRB for 20 minutes and then rinsed thrice with 1% acetic acid. Bound SRB were dissolved in 10 mM Tris, then we measured the absorbance at 540 nm.

### Lentivirus transduction and *Celigo* image cytometry assay

The PTPRO-siRNA lentivirus (shPTPRO) and negative control lentivirus (shCtrl) were obtained from Shanghai GeneChem Co., Ltd. (Shanghai, China). The SW1990 cell line was infected with lentivirus as per the instructions of the manufacturer. We used a fluorescence microscope (Olympus IX71, Tokyo, Japan) to examine the cells for presence of the GFP marker three days following infection. Subsequently, we seeded (2500 cells/well) the transfected SW1990 cells into 96-well plates and incubated them at 37 °C with 5% CO_2_ for five days. Daily counting of cell number was conducted using the Celigo^®^ Image Cytometer (Nexcelom, USA).

### Cell apoptosis

We seed the SW1990 cells (5 × 10^5^ cells/mL) into six-well plates then incubated them for 24 hours together with **GP03** compound (100μM). Subsequently, the cells were collected via trypsinization then washed two times in cold PBS. Next, the cells were centrifuged, then supernatants removed after which the cells were resuspended in 400μL of 1×binding buffer, then added to 5μL of annexin V-FITC and left to stand for 15 minutes at RT. After that, we added 10μL of PI to the cells then maintained them again at RT for 15 minutes, but in the dark. Finally, we used a flow cytometer (BD Accuri C6) to analyze the stained cells.

### Cell cycle

We seed the SW1990 cells (5 × 10^5^ cells/mL) into six-well plates then incubated them for 24 hours together with **GP03** compound (100 μM). Subsequently, the cells were collected via trypsinization then washed two times in cold PBS. Next, the cells were centrifuged, then supernatants removed after which the cells were resuspended in 400 μL of 1×binding buffer, then added to 5μL of annexin V-FITC and left to stand for 15 minutes at RT. After that, we added 10μL of PI to the cells then maintained them again for 15 minutes at RT, but in the dark. Finally, we used a flow cytometer (BD Accuri C6) to analyze the stained cells.

## Results

### Expression of PTPRO in various forms of human cancers

**Figure 1:**
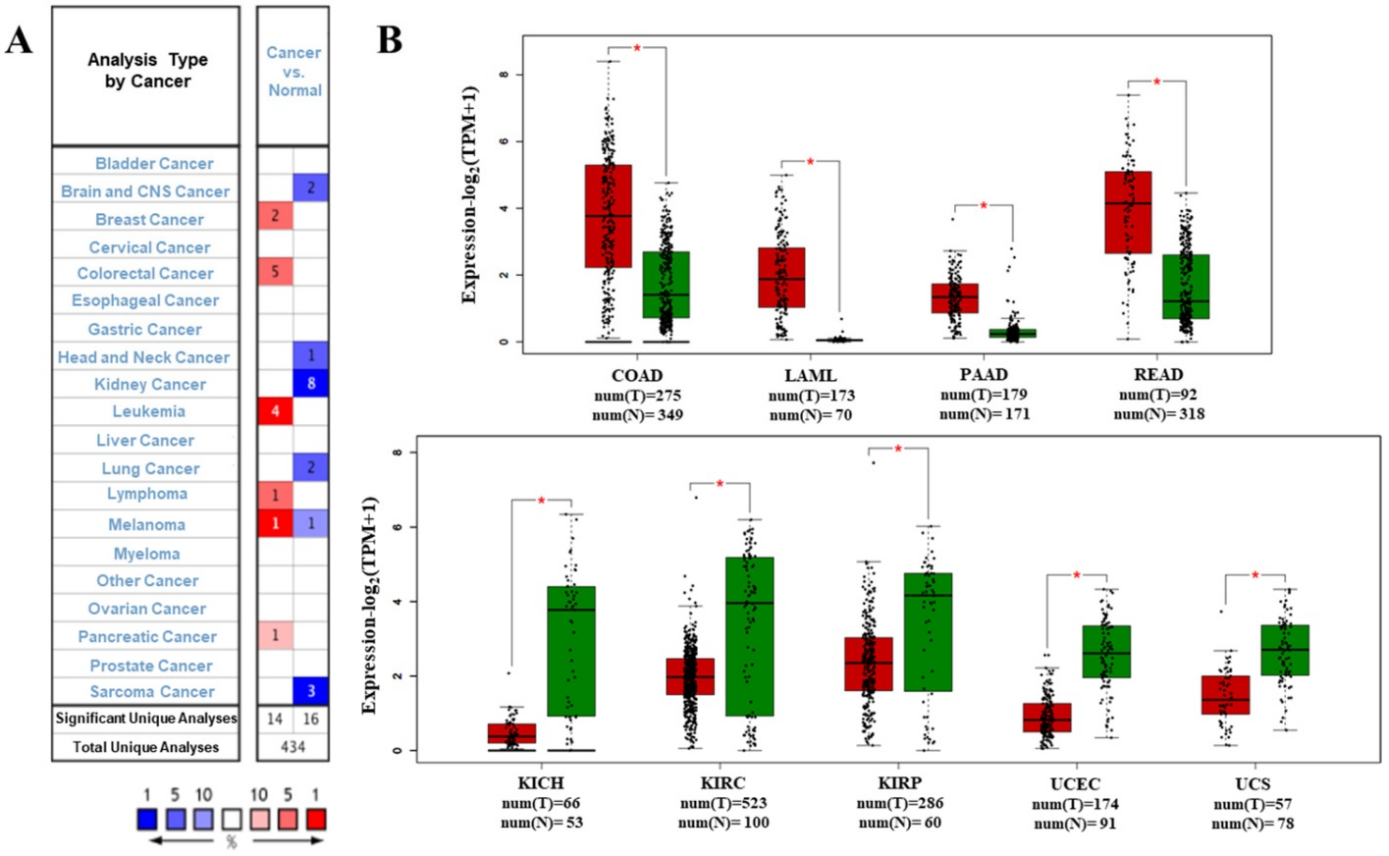
Expression of PTPRO in various forms of human cancers. **(A)** Upregulation or downregulation of PTPRO in data sets of various cancers, relative to normal tissues based on the Oncomine database. **(B)** Expression of PTPRO in various types of human cancers from TCGA database as assessed via GEPIA 25 (*P < 0.01).

We analyzed the data on the levels of PTPRO mRNA to compare the expression of PTPRO in various forms of cancers. The data which included information on tumor tissues versus their corresponding normal tissues were analyzed via the Oncomine database. According to the results, PTPRO was upregulated in breast, leukemia, lymphoma, colorectal, pancreatic cancers, and melanoma, relative to the matched normal tissues (Figure 1A). Also, downregulation of PTPRO was noted in bladder, colorectal, prostate, breast, lung, ovarian, head and neck cancers of certain data sets. We further assessed the expression of PTPRO expression in human cancers. At this stage, we employed the GEPIA server to analyze RNA-seq data of several cancers in TCGA. Significant upregulation of PTPRO was observed in colon adenocarcinoma (COAD), pancreatic adenocarcinoma (PAAD), rectum adenocarcinoma (READ), and acute myeloid leukemia (LAML), in comparison to matched normal tissues. However, PTPRO was downregulated in kidney chromophobe (KICH), kidney renal clear cell carcinoma (KIRC), uterine carcinosarcoma (UCS), uterine corpus endometrial carcinoma (UCEC), and kidney renal papillary cell carcinoma (KIRP), relative to the corresponding normal tissues (Figure 1B).

### Prognostic potential of PTPRO in human cancers

We conducted several analyses to determine whether there is a link between cancer prognosis and the expression of PTPRO. The PrognoScan was employed to assess whether PTPRO expression influence survival rates (Supplementary Table 1). Markedly, overexpression of PTPRO was associated with poorer prognosis in five type cancers, which included blood, brain, breast, esophagus and lung cancers. For example, two cohorts (GSE4475 and GSE5122) included 158 B-cell lymphoma samples and 58 AML samples revealed that PTPRO upregulation was related to poorer prognosis (OS HR = 2.02, 95% CI = 1.46 to 2.18, Cox *P* = 0.00002; OS HR = 1.62, 95% CI=1.12 to 2.34, Cox *P* = 0.01). Interesting, another cohort (GSE12417-GPL570), which include 79 AML samples, showed high PTPRO expression were associated with better prognosis (OS HR = 0.10, 95% CI= 0.02 to 0.59, Cox *P* = 0.01). In terms of breast cancer, three cohorts (E-TABM-158, GSE9195 and GSE7390) showed high PTPRO expression were linked to poorer prognosis (DMFS HR = 2.51, 95% CI = 1.27 to 4.98, Cox* P* = 0.008; RFS HR = 3.36, 95% CI=1.03 to 10.97, Cox *P* = 0.04; DMFS HR = 1.41, 95% CI = 1.00 to 1.98, Cox *P* = 0.05), however, one cohort (GSE7849) showed high PTPRO expression were related to better prognosis (DFS HR = 0.56, 95% CI = 0.33 to 0.95, Cox *P* = 0.03). Although we observed higher expression of PTPRO in colorectal cancer compared with normal samples (Figure 1B), two cohorts (GSE17537 and GSE17537) indicated that the high expression of PTPRO seems to be associated with better prognosis (OS HR = 0.57, 95% CI = 0.36 to 0.91, Cox *P* = 0.02; DFS HR = 0.56, 95% CI=0.34 to 0.93, Cox *P* = 0.03).

We further employed the Kaplan-Meier plotter database to evaluate the prognostic significance of PTPRO in pancreatic and rectum cancer samples, which are not included in the PrognoScan database. Notably, poor prognosis of pancreatic (OS HR = 1.31, 95% CI = 0.86 to 2.00, P = 0.2; RFS HR = 4.23, 95% CI = 1.17 to 15.31, P = 0.02) correlated with PTPRO overexpression. However, PTPRO expression was linked to a better OS and RFS in rectum cancer. These results suggested that PTPRO upregulation could be an independent risk factor for poor disease outcomes in patients of pancreatic cancer. The results above revealed that PTPRO expression affects the prognosis of specific types of cancer.

### Anti-proliferation activity of PTPRO inhibition

**Figure 4:**
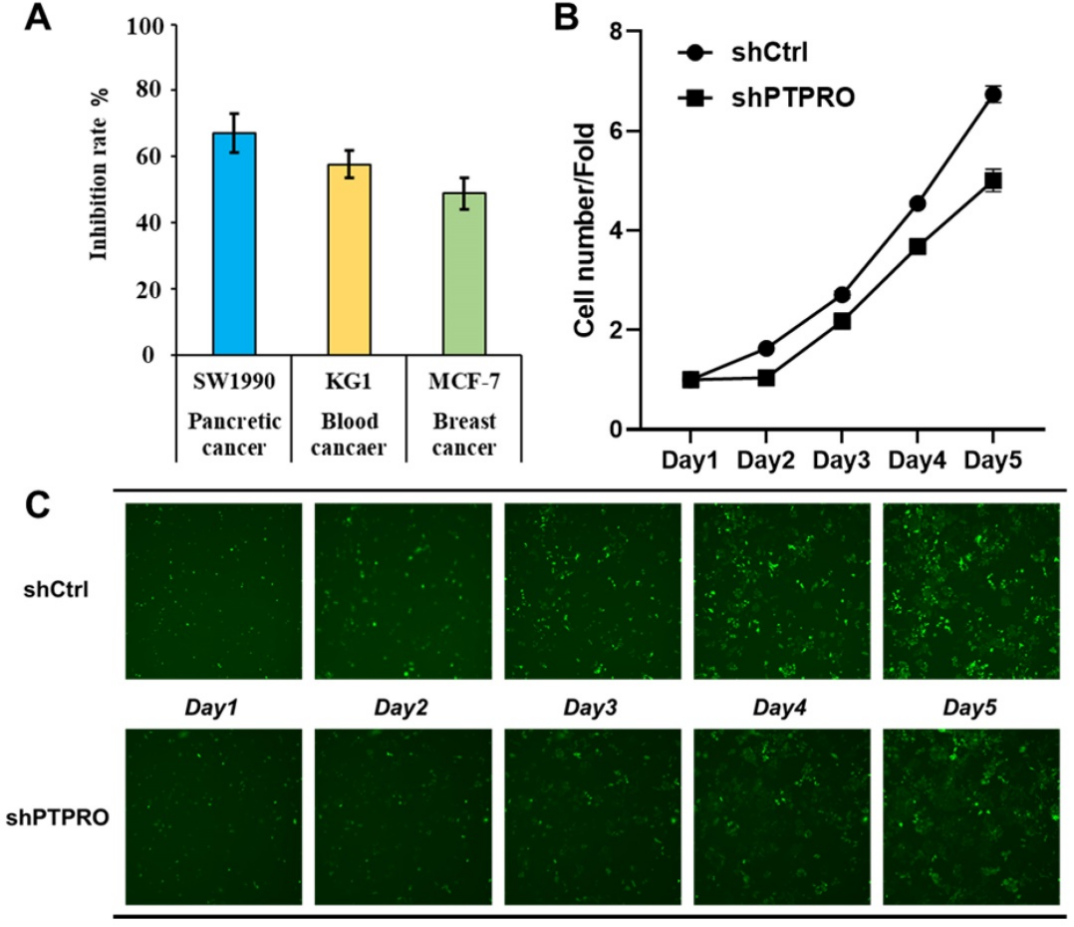
** (A)** The anti-proliferation activities of PTPRO inhibitor **GP03** at 50μM against three cancer cell lines. **(B)** Effects of PTPRO knock-down on proliferation of pancreatic cancer cells. **(C)** Cell counts using the *Celigo* system (×100 magnification).

Bioinformatic analysis above suggested the potential oncogenic role of PTPRO in blood, breast, and pancreatic cancers. In our previous work, a selective PTPRO inhibitor **GP03** has been identified through structure-based virtual screening (Figure S1 in Supporting Information) 26. Therefore, we measured the anti-proliferation activities of **GP03** against three cancer cell lines using sulforhodamine B assay (SRB) assay, including KG1 cells (blood cancer), MCF-7 cells (breast cancer) and SW1990 cells (pancreatic cancer). As results shown in Figure 4A, **GP03** exhibited anti-proliferation activity against three type of cancer cells, of which the anti-proliferation activity on pancreatic cancer cells is the strongest. Moreover, **GP03** inhibited the growth of SW1990 cells in a dose-dependent manner (Figure S2 in Supporting Information). To confirm the oncogenic role of PTPRO, we knocked-down the expression of PTPRO in pancreatic cancer cells using lentiviral and measured the cell viability using *Celigo* and flow cytometry assays, which based on GFP-expressing cancer cells. As expected, knock-down of the expression of PTPRO inhibited growth of pancreatic cancer cell.

### Effects of PTPRO inhibitor on cell apoptosis and cell cycle

**Figure 5:**
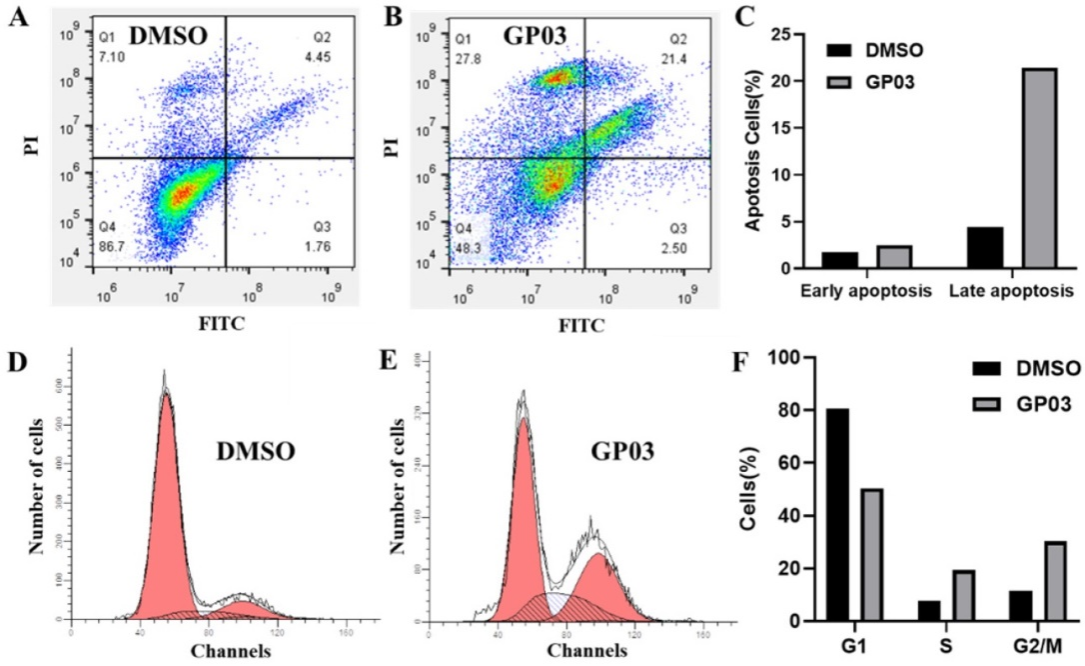
** (A-C)** Apoptosis induction of pancreatic cancer cells by DMSO and PTPRO inhibitor **GP03** at 100 μM. **(D-F)** Cell cycle distribution of pancreatic cancer cells treated with DMSO and PTPRO inhibitor **GP03** at 100 μM.

Here, we further evaluated the effects of PTPRO inhibitor **GP03** on cell apoptosis as well as cell cycle of pancreatic cancer cells. We conducted the Annexin V-FITC/PI assay to determine the apoptotic induction effect of **GP03**. According to the results, compound **GP03** effectively induced apoptosis of pancreatic cancer cells, compared with DMSO control (Figure 5A-C). Furthermore, cell cycle distribution of pancreatic cancer cells was evaluated following DMSO and **GP03** treatment. Based on the results, the progression of cell cycle was blocked at the G2 and S stages 24 hours following compound **GP03** treatment (Figure 5D-F).

### Correlation of PTPRO expression with clinical prognosis and immune cell enrichment levels

Kaplan-Meier plotter database was employed to examine the link between PTPRO expression and clinical features, as well as immune cell enrichment levels of pancreatic cancer patients. As result shown in Table 1, high levels of PTPRO mRNA was significantly related to worse OS in grade 1 pancreatic cancer patients (*P* <0.001). Moreover, upregulation of PTPRO was related to worse PFS in male patients (*P* = 0.02), grade 2 patients (*P* = 0.027) and patient with high mutation burden (*P* = 0.004).

Furthermore, we observed associations of PTPRO expression with worse OS and PFS in patients with enriched CD8+ T-cells level (OS, *P* = 0.026; PFS, *P* = 0.008) and decreased macrophages level (OS, *P* = 0.013; PFS, *P* = 0.0072). The enrichment levels of B-cells and CD4+ memory T-cells show less impacts on the prognosis values (OS), where high expressions of PTPRO are associated with worse PFS. In addition, overexpression of PTPRO was also linked to worse PFS values in patients with enriched eosinophils level (*P* = 0.0053) or enriched regulatory T-cells level (*P* = 0.021) (Table 2). These data indicated that the level of PTPRO expression affect the prognosis of pancreatic cancer patient having specific clinicopathological characteristics and immune cell enrichment levels.

### GSEA analysis revealed pathways associate with PTPRO expression in pancreatic cancers

To further elucidate the function of PTPRO in pancreatic cancer, Gene Set Enrichment Analyses (GSEA) we carried out using the TCGA dataset. Single gene GSEA analyses indicate that the high expression of PTPRO was obviously enriched in the expression of gene signatures associated with cytokine-cytokine receptor interaction, natural killer (NK) cell-mediated cytotoxicity, and Jak-STAT signaling pathway. In particular, the Jak-STAT signaling pathway controlling cellular processes including proliferation, differentiation and apoptosis 27, 28, which may explain the anti-cancer activity of PTPRO inhibitor. Furthermore, the GESA result also revealed the potential role of PTPRO in tumor immunology. Cytokines has been used to direct immune effector cells to directly attack and destroy the tumor cells 29. Pro-inflammatory cytokines have been found to exhibit potent anti-tumour activities in animal models. Evidence show that NK cells quickly kill close by cells that exhibit surface markers related to oncogenic transformation. This activity of NK cells is unique amongst the immune cells. Also, NK cells play their anticancer role by facilitating the responses of antibody and T-cells 30.

### PTPRO expression is linked to infiltration level of immune cells in pancreatic cancers

Inspired by the results of analysis above, we speculated that PTPRO expression might be related to infiltration level of immune cells in pancreatic cancers. As such, we used the TIMER to explore this hypothesis. Based on the results, PTPRO expression showed an inverse relationship with tumor purity (*r* = -0.324, *P* = 1.45e-05) in pancreatic cancers. Notably, PTPRO expression was positively related to the levels of CD8^+^ T cell infiltration (*r* = 0.668, *P* = 1.78e-23), neutrophils (*r* = 0.73, *P* = 1.00e-29), macrophages (*r* = 0.519, *P* = 3.45e-13) and dendritic cells (*r* = 0.817, *P* = 2.44e-42) in pancreatic cancers. The CD8^+^ T cells are preferred immune cells for cancer immunotherapy with the ability to detect and eradicate cancer cells 31. Neutrophils and macrophages can be divided into different subsets and possess either antitumorigenic or protumorigenic functions 32, 33. Dendritic cells initiate and regulate adaptive immune responses, thereby harness the ability of the immune system to recognize and eliminate cancer 34. On the other hand, the expression of PTPRO indicated a weak correlation with CD4^+^ T cells (*r* = 0.031, *P* = 6.84e-01) and B cells (*r* = 0.025, *P* = 7.45e-01) in pancreatic cancers. These data indicated that PTPRO could play a precise function in infiltration of immune cells (specifically, neutrophils and dendritic cells) with regards to pancreatic cancers.

## Discussion

In this manuscript, we systematically investigated the differential expression of PTPRO as well as its prognosis values in different human cancers. Bioinformatic analysis revealed the potential oncogenic role of PTPRO in several cancer type, especially pancreatic cancer. Upregulation of PTPRO is related to a poorer prognosis in PAAD. Thereafter, *in vitro* tests proved that inhibition of PTPRO effected the growth of pancreatic cancer cells. Moreover, small molecular PTPRO inhibitor **GP03** not only induced cell apoptosis of pancreatic cancer cells, but also inhibited cell cycle progression in G2 and S phase. Further GSEA analysis revealed several signaling pathways associate with PTPRO expression, which helps to understand the underlying carcinogenic functions of PTPRO in pancreatic cancer. Specially, the two most relevant pathways play vital roles in tumor immunology. In addition, the expression of PTPRO exhibited strong relevance with tumor-infiltrating immune cells, which suggested the potential role of PTPRO in anti-tumor immunity. Taken together, our findings indicated that inhibition of PTPRO may serve as a new strategy for the treatment of pancreatic cancer.

## Supplementary Material

Supplementary figures and tables.

## Figures and Tables

**Figure 2:**
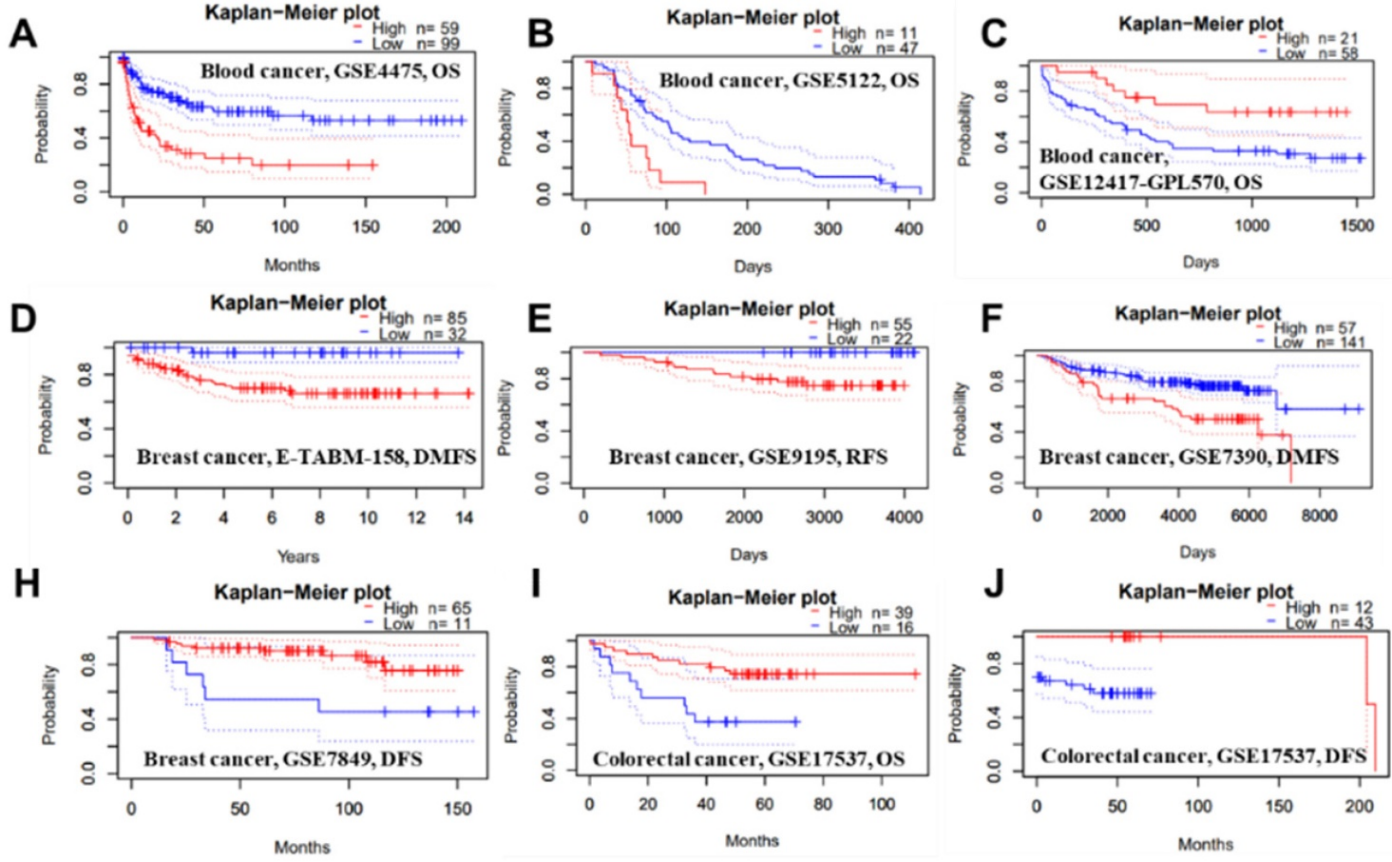
Representative Kaplan-Meier survival curves showing the high versus low PTPRO expression in various forms of cancer based on the PrognoScan databases.

**Figure 3:**
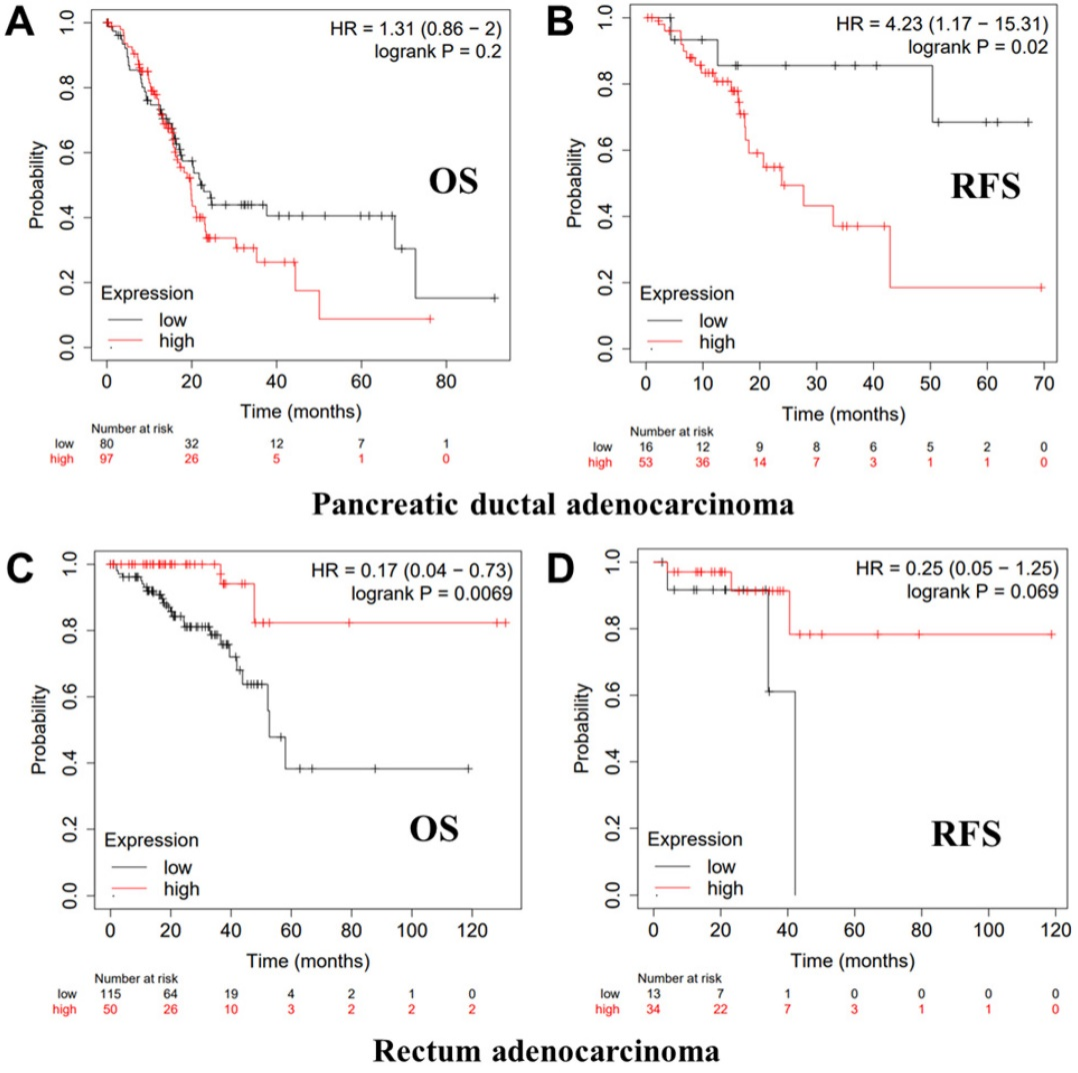
Kaplan-Meier survival curves showing the high versus low PTPRO expression in various cancer types based on the Kaplan-Meier plotter databases.

**Figure 6:**
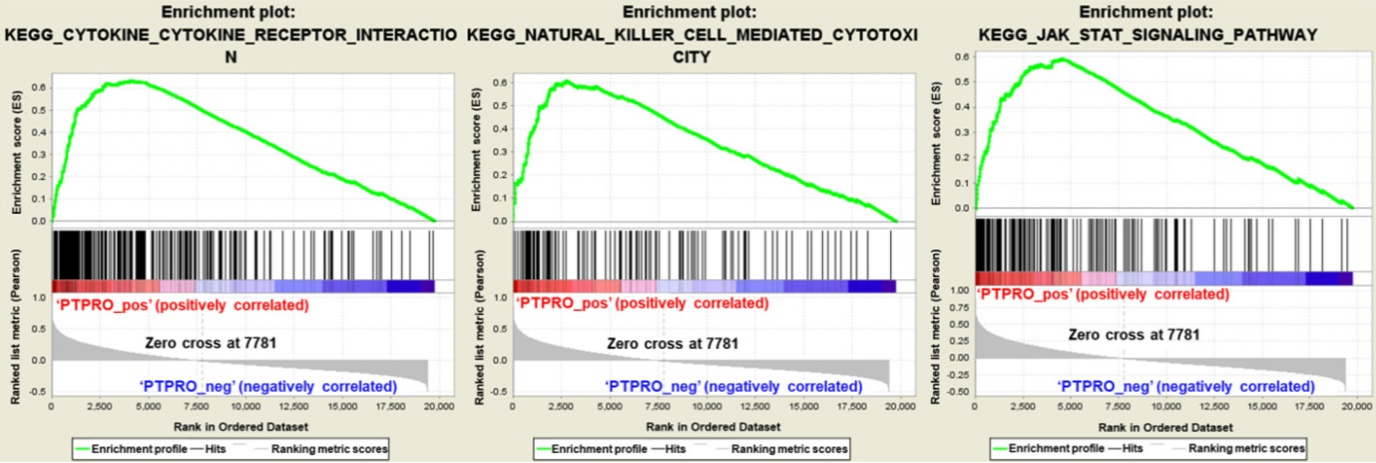
Gene set enrichment analysis in pancreatic cancer (PAAD) based on the Kyoto Encyclopedia of Genes and Genomes (KEGG) pathways. PAAD samples were correlated positively with gene signatures that are linked to cytokine-cytokine receptor interaction, natural killer cell mediated cytotoxicity, and Jak-STAT signaling pathway.

**Figure 7:**
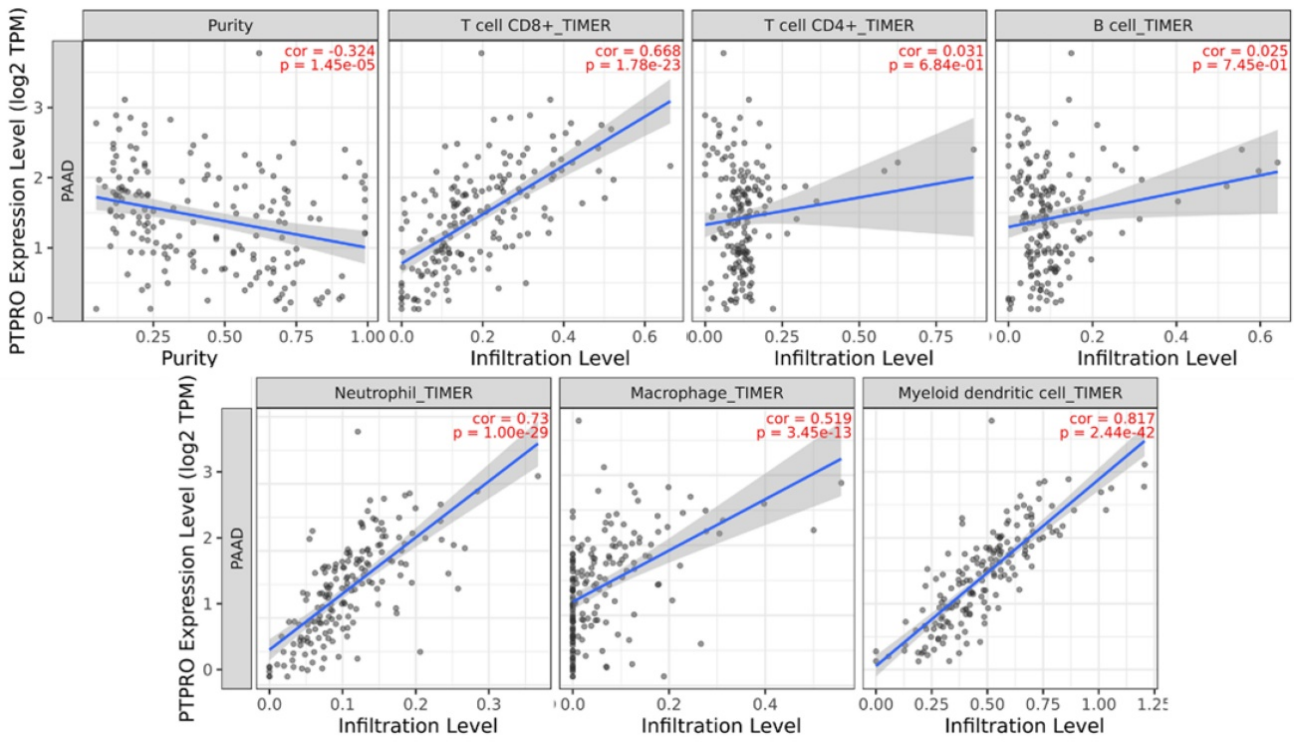
PTPRO expression versus immune infiltration level in PAAD (pancreatic adenocarcinoma).

**Table 1:** Correlation of PTPRO mRNA expression and clinical prognosis in pancreatic cancer with different clinicopathological factors by Kaplan-Meier plotter database

Clinicopathological characteristics	OS (n=177)			PFS (n=69)		
	N	Hazard ratio	*P*-value	N	Hazard ratio	*P*-value
**Sex**						
Female	80	1.67(0.89-3.16)	0.11	32	1.91(0.5-7.23)	0.34
Male	97	0.72(0.37-1.39)	0.32	37	NA	**0.02`**
**Stage**						
1	21	NA	0.097	NA	NA	NA
2	146	0.73(0.45-1.18)	0.19	55	2.25(0.86-5.88)	0.088
3	NA	NA	NA	NA	NA	NA
4	NA	NA	NA	NA	NA	NA
**Grade**						
1	31	14.68(1.85-116.73)	**0.00099**	NA	NA	NA
2	94	0.69(0.38-1.26)	0.23	38	0.29(0.09-0.93)	**0.027**
3	48	0.62(0.28-1.37)	0.23	NA	NA	NA
4	NA	NA	NA	NA	NA	NA
**Mutation burden**						
High	84	1.66(0.91-3.01)	0.093	29	6.58(0.82-52.99)	**0.044**
Low	83	1.51(0.71-3.21)	0.28	33	3.55(0.43-29.3)	0.21

NA: Data not available because sample number too low for meaningful analysis.

**Table 2:** Correlation of PTPRO mRNA expression and clinical prognosis in pancreatic cancer with different levels of immune cell enrichment by Kaplan-Meier plotter database

Levels of immune cell enrichment		OS (177)			PFS (69)		
		N	Hazard ratio	*P*-value	N	Hazard ratio	*P*-value
Basophils	Enriched	NA	NA	NA	NA	NA	NA
	Decreased	164	0.75 (0.49-1.14)	0.18	62	1.62 (0.64-4.08)	0.31
B-cells	Enriched	59	1.63 (0.67-4.02)	0.28	21	9.67 (0.93-100.05)	**0.024**
	Decreased	118	1.32 (0.79-2.19)	0.28	48	6.84 (1.5-31.11)	**0.0051**
CD4+ memory T-cells	Enriched	43	0.48 (0.16-1.48)	0.19	20	NA	**0.011**
	Decreased	134	1.36 (0.81-2.28)	0.25	49	11.01 (1.44-84.19)	**0.0043**
CD8+ T-cells	Enriched	76	2.22 (1.08-4.53)	**0.026**	35	NA	**0.008**
	Decreased	101	1.23 (0.73-2.07)	0.44	34	4.39 (0.55-34.84)	0.13
Eosinophils	Enriched	154	1.34 (0.79-2.25)	0.28	60	6.98 (1.52-32.05)	**0.0053**
	Decreased	23	0.51 (0.16-1.56)	0.23	NA	NA	NA
Macrophages	Enriched	109	1.43 (0.84-2.43	0.19	46	2.1 (0.73-6.01)	0.16
	Decreased	68	2.98 (1.21-7.35)	**0.013**	23	10.91 (1.26-94.76)	**0.0072**
Mesenchymal stem cells	Enriched	156	0.8 (0.49-1.3)	0.37	58	1.89 (0.74-4.82)	0.17
	Decreased	21	2.42 (0.53-11.05)	0.24	NA	NA	NA
Natural killer T-cells	Enriched	60	2.87 (1.16-7.12)	**0.017**	21	4.29 (0.44-42.16)	0.18
	Decreased	97	1.46 (0.85-2.52)	0.17	48	2.3 (0.87-6.1)	0.086
Regulatory T-cells	Enriched	67	2.17 (0.91-5.14)	0.073	25	NA	**0.021**
	Decreased	110	0.7 (0.41-1.19)	0.19	44	5.73 (0.72-45.45)	0.065
Type 1 T-helper cells	Enriched	NA	NA	NA	NA	NA	NA
	Decreased	165	1.27 (0.82-1.95)	0.28	63	2.49 (0.78-7.95)	0.11
Type 2 T-helper cells	Enriched	41	1.6 (0.75-3.4)	0.22	NA	NA	NA
	Decreased	146	1.6 (0.94-2.73)	0.079	51	3.79 (0.82-17.48)	0.068

NA: Data not available because sample number too low for meaningful analysis.
