# Supplementary material for: PTPRO is a therapeutic target and correlated with immune infiltrates in pancreatic cancer
Source: J Cancer. 2021 Oct 30;12(24):7445–53. doi: 10.7150/jca.64661 (PMC8734421; doi:10.7150/jca.64661)
Supplement: Supplementary file 1 — Supplementary figures and tables. [file jcav12p7445s1.pdf]

PTPRO is a therapeutic target and correlated with immune infiltrates in pancreatic cancer

Xuben Hou [1], Jintong Du [2,*] and Hao Fang [1,*]

[1] Department of Medicinal Chemistry and Key Laboratory of Chemical Biology of Natural Products (MOE), Cheeloo College of Medicine, School of Pharmaeutical Science, Shandong University, Jinan, Shandong, 250012, China

[2] Shandong Cancer Hospital and Institute, Shandong First Medical University, Jinan, Shandong, 250117, China

Supplementary Materials

Table S1. Relation between PTPRO expression and patient prognosis of different cancer in Prognoscan database (Cox *P*-value < 0.05).

Cancer type	Database	Endpoint	N	Cox *P*-value	ln(HR)	HR [95% CI^{low} - CI^{upp}]
Blood cancer	GSE4475	OS	158	0.000024	0.71	2.02 [1.46 - 2.81]
Blood cancer	GSE12417-GPL570	OS	79	0.010492	-2.29	0.10 [0.02 - 0.59]
Blood cancer	GSE5122	OS	58	0.011185	0.48	1.62 [1.12 - 2.34]
Brain cancer	GSE16581	OS	67	0.002598	2.46	11.72 [2.36 - 58.14]
Breast cancer	E-TABM-158	DMFS	117	0.008087	0.92	2.51 [1.27 - 4.98]
Breast cancer	GSE7849	DFS	76	0.031508	-0.59	0.56 [0.33 - 0.95]
Breast cancer	GSE9195	RFS	77	0.044722	1.21	3.36 [1.03 - 10.97]
Breast cancer	GSE7390	DMFS	198	0.048062	0.34	1.41 [1.00 - 1.98]
Colorectal cancer	GSE17537	OS	55	0.018226	-0.55	0.57 [0.36 - 0.91]
Colorectal cancer	GSE17537	DFS	55	0.026472	-0.58	0.56 [0.34 - 0.93]
Esophagus cancer	GSE11595	OS	34	0.040433	1.03	2.79 [1.05 - 7.44]
Esophagus cancer	GSE11595	OS	34	0.040433	1.03	2.79 [1.05 - 7.44]
Lung cancer	GSE31210	RFS	204	0.004968	0.7	2.02 [1.24 - 3.29]
Lung cancer	GSE31210	OS	204	0.013116	0.83	2.30 [1.19 - 4.44]
Lung cancer	jacob-00182-MSK	OS	104	0.037743	-1.02	0.36 [0.14 - 0.94]
Ovarian cancer	GSE9891	OS	278	0.03363	-0.27	0.76 [0.60 - 0.98]
Soft tissue cancer	GSE30929	DRFS	140	0.021113	-0.4	0.67 [0.48 - 0.94]

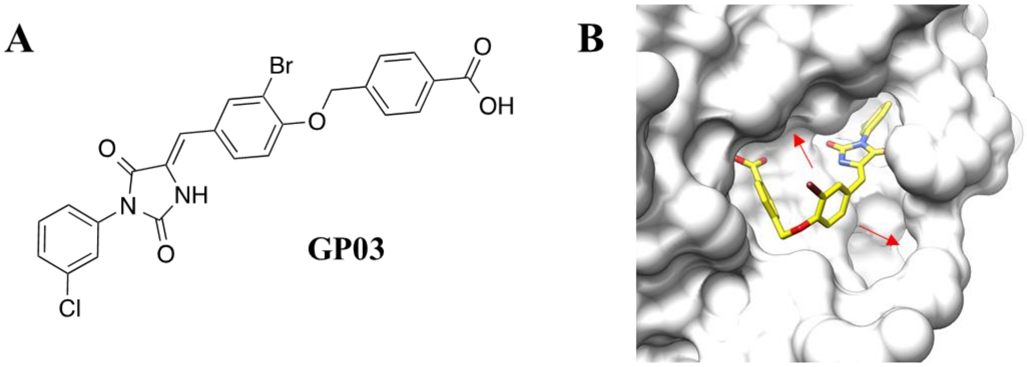

Figure S1. (A) Chemical structure of PTPRO inhibitor **GP03**. (B) Predicted binding mode of **GP03** against PTPRO. Molecular docking was performed using Autodock Vina. The unoccupied spaces were marked with red arrows.

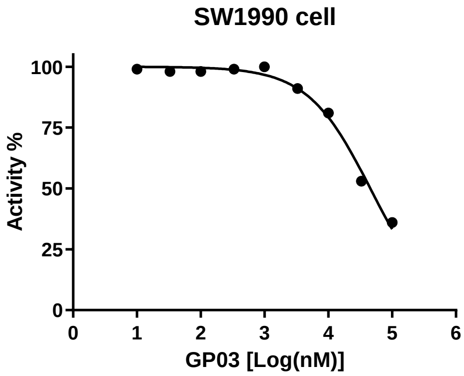

Figure S2. Dose-dependent inhibition of **GP03** on proliferation of SW1990 cells.
